# Harnessing photography and image recognition technology to aid in the elimination of trachoma

**Published:** 2022-06-07

**Authors:** Cristina Jimenez, Emily Gower, Emma Harding-Esch, Sheila K West

**Affiliations:** 1 Tropical Data Programme Manager: Sightsavers, Haywards Heath, UK.; 2 Associate Professor: University of North Carolina, Chapel Hill, USA.; 3 Tropical Data Chief Scientist and Associate Professor: London School of Hygiene & Tropical Medicine, London, UK.; 4 El-Maghraby Professor: Johns Hopkins Medicine, Atlanta, USA.

**Technology can help to overcome human resource challenges in active trachoma grading and case finding for trachomatous trichiasis.**

Significant progress has been made to reduce the global burden of trachoma in recent years. Since 2002, the number of people at risk has reduced by 92%, from 1.5 billion to 124 million. The number of people estimated to require surgery to treat trachomatous trichiasis (TT), the late blinding stage of trachoma, has reduced by 76% in the same period: from 7.6 million to 1.8 million.

When it comes to training people to grade active trachoma, or find people who need TT surgery (known as TT case finding), however, the global trachoma programme is becoming a victim of its own success. As the prevalence of trachoma declines, it is increasingly difficult to find individuals with active trachoma or TT who can serve as examples during the training of new field graders, or when re-certifying existing graders. This makes training more difficult, expensive and time consuming, and represents a significant challenge when integrating trachoma interventions into routine health services. Additionally, there are situations where the use of human case-finders or trained graders may not be ideal or sustainable, such as in conflict zones (where graders may not be allowed) or in remote or hard-to-reach areas.

A recent report^1^ presents results of a series of workshops summarising existing studies and exploring the opportunities, challenges, and future operational research priorities related to the use of photography and image grading for trachoma. The report includes data from a systematic review on the utility of photography for trachoma surveys, and the development and testing of metrics for assessing the quality of images. It also highlights the inclusion of 3D images that provide greater specificity for TT diagnosis. These have been routinely included in trachoma training systems supported by Tropical Data^2^ – a consortium of partners supporting health ministries to conduct globally standardised, high quality trachoma prevalence surveys that conform with World Health Organization recommendations. Results from studies seeking to replace field grading included the use of a head-mounted ‘Image Capture and Processing System’ (ICAPS) piloted in Tanzania and the development of a photography grading centre to increase local capacity for assessing images in Ethiopia.

Many opportunities and challenges were identified related to the use of photography to support training, supervision, and field grading in the future. These include the development of a library of appropriate images for training purposes, and establishing a consensus grading method for photographic images. Diverse options for photograph grading to replace field grading were explored, as was ensuring the feasibility of these new approaches. Different working groups organised by members of the trachoma community are now working to address these issues through the pursuit of various operational research projects.

**Figure:**
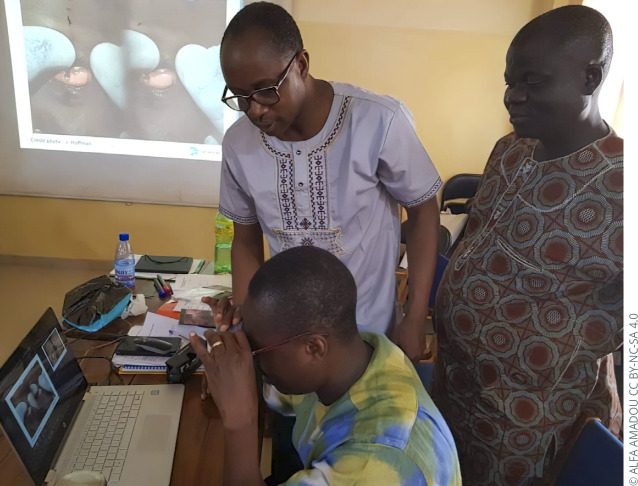
Classroom learning for TT-only surveys using 3D images. **BENIN**

Researchers at the University of North Carolina at Chapel Hill and RTI International are carrying research into an image recognition algorithm and an associated smartphone app that identifies TT in eyelids using machine learning techniques. The user-friendly app allows users to enter participant information and take a photograph. After the photograph is taken, the algorithm determines whether the eye has TT. At present, this technology is showing positive results and is being pilot tested with Tropical Data support. In the coming months, the algorithm and app will be tested more broadly with a large group of TT case-finders in Senegal.

To achieve the global elimination of trachoma as a public health problem, it will be critical to overcome human resource challenges, including grading for active trachoma and case-finding for TT. Technology offers a real opportunity to address the challenges of TT case finding and trachoma grading in a cost-effective way. However, there is currently a lack of standardisation and validated protocols outside of research projects for the use of photographs to train graders, for supervision purposes, and to serve as an adjunct to, or substitute for, field grading. Resolving these challenges will be critical if we are to scale up these new technologies and achieve vision for all.

## References

[1] Use of photography for support of trachoma grading: Progress report. 2022. International Coalition for Trachoma Control https://bit.ly/3lPzIRj

[2] Tropical Data.. https://bit.ly/3Gpj8RS.

